# Lower levels of proteinuria are associated with elevated mortality in incident dialysis patients

**DOI:** 10.1371/journal.pone.0226866

**Published:** 2019-12-23

**Authors:** Manabu Hishida, Tariq Shafi, Lawrence J. Appel, Shoichi Maruyama, Daijo Inaguma, Kunihiro Matsushita

**Affiliations:** 1 Department of Epidemiology, Johns Hopkins Bloomberg School of Public Health, Baltimore, MD, United States of America; 2 Department of Nephrology, Graduate School of Medicine, Nagoya University, Nagoya, Japan; 3 Johns Hopkins University School of Medicine, Baltimore, MD, United States of America; 4 Department of Nephrology, Fujita Health University Hospital, Toyoake, Aichi, Japan; International University of Health and Welfare, School of Medicine, JAPAN

## Abstract

**Introduction:**

Proteinuria is a potent predictor of adverse events in general, although a few large studies have reported a J-shaped association between proteinuria and mortality in individuals with glomerular filtration rate <30 ml/min/1.73m^2^. However, this association has not been specifically evaluated among incident dialysis patients.

**Methods:**

Among 1,380 Japanese patients who initiated dialysis, we quantified the association of pre-dialysis dipstick proteinuria (negative/trace, 1+, 2+, and ≥3+) with mortality using Cox models adjusting for potential confounders, such as age, gender, clinical history of hypertension, diabetes, and cardiovascular disease.

**Results:**

Mean age of study participants was 67.4 (SD 13.0) years, and 67.6% were men. The most common dipstick proteinuria category was ≥3+ (55.4%), followed by 2+ (31.2%), 1+ (9.9%), and negative or trace (3.5%). Patients with lower proteinuria level were older than those with higher proteinuria. Lower proteinuria was significantly associated with a higher risk of all-cause mortality, even after accounting for potential confounders (p for trend <0.001). In those with negative/trace dipstick proteinuria compared to those with dipstick proteinuria ≥3+, the adjusted hazard ratio was 2.60 [95% CI: 1.62–4.17] in the fully adjusted model. Similar findings were observed when analyses were restricted to patients older than 70 years, and when cardiovascular mortality and non-cardiovascular mortality were analyzed separately.

**Conclusions:**

In incident dialysis patients, pre-dialysis proteinuria was inversely associated with mortality risk. Although future studies are needed to identify mechanisms, our findings suggest the need to carefully interpret proteinuria in patients with incident dialysis.

## Introduction

Increased levels of proteinuria are a potent predictor of several adverse health outcomes such as mortality, cardiovascular disease, and end-stage renal disease [1–4]. As a consequence, the KDIGO 2012 Chronic Kidney Disease (CKD) Clinical Practice Guideline recommends characterizing CKD according to both glomerular filtration rate (GFR) and proteinuria [5].

Although proteinuria has been shown as a prognostic factor in a wide range of populations [6–8], a few studies have shown counterintuitive associations between proteinuria and mortality among patients with severely reduced GFR <30 ml/min/1.73m^2^. For example, an international individual-level meta-analysis from the CKD Prognosis Consortium including 14 cohorts demonstrated a J-shaped association between proteinuria and all-cause mortality in GFR 15–29 ml/min/1.73m^2^, with the lowest risk in urine albumin-to-creatinine ratio 10–29 mg/g and a higher risk in albuminuria <10 mg/g in addition to albuminuria ≥30 mg/g [6]. This pattern was even enhanced for cardiovascular mortality, with its highest risk observed in albuminuria <10 mg/day in this GFR category [6]. Importantly, a J-shaped pattern in GFR <30 ml/min/1.73m^2^ was also documented in a large study of US veterans [9].

However, the association between proteinuria and adverse outcomes has not been studied among patients with incident dialysis. Therefore, we evaluated this association among Japanese patients with incident dialysis. We also explored whether the associations are consistent for cardiovascular mortality and non-cardiovascular mortality.

## Materials and methods

### Study population

We used data from the Aichi Cohort Study of Prognosis in Patients Newly Initiated into Dialysis (AICOPP), including 1,520 incident dialysis patients. Details of AICOPP were described previously [10]. Briefly, this cohort recruited patients who initiated dialysis between October 2011 and September 2013 at 17 facilities in Aichi, Japan. We screened patients aged at least 20 years and then enrolled who were discharged alive from hospitalization for dialysis initiation. Each patient provided written consent. For the current study, of 1,520 patients in AICOPP, we excluded 24 patients with missing values of proteinuria and 116 patients with missing values of covariates of interest, leaving a final study sample of 1,380 patients (S1 Fig).

### Baseline variables

Baseline demographic and clinical data, including blood test and urine test were collected just before or during the hospitalization for dialysis initiation. Proteinuria was semi-quantitatively assessed by dipstick strips and classified into four categories (negative/trace, 1+, 2+, and ≥3+) [4]. Dipsticks were not necessarily the same among facilities, but the readings were consistently done by machine in all facilities. The protein concentrations corresponding to negative/trace, 1+, 2+, and 3+ were <30mg/dL, ≥30mg/dL, ≥100mg/dL, ≥300mg/dL. Estimated GFR (eGFR) was calculated using the Japanese Society of Nephrology’s equation (eGFR = 194 × serum creatinine—1.094 × age—0.287 [×0.739 for women]) [11]. Diabetes mellitus was defined by hemoglobin A1c ≥6.5%, use of antidiabetic medications, or prior diagnosis of diabetes. A clinical history of coronary artery disease requiring revascularization, heart failure requiring hospitalization, or stroke was reported by facility physicians. Primary kidney disease for each patient was determined by a nephrologist based on renal biopsy, imaging modalities such as ultrasound, patient characteristics (e.g., age, family history, comorbidities), and clinical progress. Blood pressure was measured at the start of first dialysis therapy. Body mass index (BMI) was calculated from pre-hemodialysis weight in kilograms, divided by the square of height in meters (kg/m^2^). Performance in activities of daily living was evaluated using Barthel index [12]. The use of diuretics before dialysis initiation included any regular use of loop diuretics, thiazide-type diuretics, or spironolactone. The use of angiotensin-converting-enzyme inhibitors or angiotensin receptor blockers was defined as regular use of these medicines before dialysis. Ejection fraction was obtained through the echocardiography.

### Outcomes

The primary outcome was all-cause mortality after initiation of dialysis. Secondary outcomes were cardiovascular disease (CVD) mortality and non-CVD mortality. AICOPP investigators contacted each dialysis clinic annually to assess survival of AICOPP participants. When deaths were reported, information on date and cause of death were collected. CVD deaths were defined as deaths due to coronary artery disease, stroke, heart failure, aortic diseases, or sudden cardiac death. Patients were followed until death, loss to follow-up, kidney transplantation, recovery from dialysis therapy, or end of follow-up on September 30, 2016 (i.e., administrative censoring).

### Statistical analysis

Baseline characteristics were summarized as mean (SD) and proportions as appropriate across the four dipstick proteinuria categories (negative/trace, 1+, 2+, and ≥3+) [4]. Differences in variables across the categories were assessed by ANOVA test. We first estimated survival across proteinuria categories using the Kaplan-Meier method. The difference in survival estimates across dipstick categories was assessed using the log-rank test. Subsequently, we explored the impact of potential confounders using multivariable Cox proportional hazards models. We explored four models: Model 1 was unadjusted; Model 2 adjusted for age and gender; Model 3 additionally accounted for a clinical history of diabetes, coronary artery disease, heart failure, and stroke; Model 4 further adjusted for systolic blood pressure (continuous), total cholesterol (continuous), eGFR, serum albumin, hemoglobin, white blood cell, use of diuretics, and angiotensin-converting-enzyme inhibitor/angiotensin receptor blockers prior to dialysis initiation. We selected proteinuria category ≥3+ as a reference because this category was most prevalent in our study population as described subsequently. P for trend was calculated by modeling dipstick proteinuria as a continuous variable of 0, 1, 2, and 3 for negative/trace, 1+, 2+, and ≥3+, respectively.

We conducted several sensitivity analyses. First, since most patients with negative/trace dipstick proteinuria were aged 70 years or older and had nephrosclerosis as primary kidney disease, we restricted our analysis to patients aged ≥70 years (there were only two deaths among 12 patients younger than 70 with negative/trace proteinuria, and thus we could not meaningfully analyze younger patients) and those with nephrosclerosis. Second, we further adjusted for ejection fraction. Since there were 238 patients with missing data of ejection fraction, this model included 1,142 patients. Finally, stratified analyses were performed for several demographic and clinical factors: gender, BMI (< vs. ≥22 kg/m^2^), diabetic status, presence or absence of a history of CVD (including coronary artery disease, heart failure, and stroke), systolic blood pressure level (< vs. ≥130 mmHg) [13], ejection fraction (<50% vs. ≥50%). The interactions between proteinuria and stratifying factors were evaluated by likelihood-ratio tests. In all analysis, a two-sided p-value less than 0.05 was considered statistically significant. All statistical analyses were performed using Stata version 14 (Stata Corp, College Station, Texas, USA).

### Statement of ethics

In this study, we followed the Japanese Ministry of Health, Labor, and Welfare’s ‘‘ethical guidelines for clinical research” (issued on July 30, 2003, and revised on December 28, 2004, and July 31, 2008) and the Helsinki Declaration. The present study was approved by the Institutional Review Boards of Fujita Health University (approval no. HM18-230), with an exemption from informed consent.

## Results

### Baseline characteristics

Mean age of 1,380 study participants at dialysis initiation was 67.4 (SD 13.0) years, and 67.6% were male. The leading cause of dialysis was diabetic nephropathy (43.5%), followed by nephrosclerosis (25.2%) and chronic glomerulonephritis (14.8%). The most common dipstick proteinuria category was ≥3+ (n = 765, 55.4%), followed by 2+ (n = 430, 31.2%) and 1+ (n = 137, 9.9%). There were 48 patients (3.5%) with proteinuria negative or trace.

Patients with lower proteinuria level were likely to be older and to have lower BMI, Barthel index, blood pressure, and ejection fraction and higher prevalence of prior coronary artery disease or heart failure (Table 1). 75.0% of patients with negative/trace dipstick proteinuria were aged 70 years or older. Diabetic nephropathy was the most common primary kidney disease in dipstick proteinuria ≥3+, whereas nephrosclerosis was the most common primary disease in dipstick proteinuria 1+ and negative/trace. The proportion of diabetic nephropathy and nephrosclerosis was similar in dipstick proteinuria 2+.

**Table 1:** Baseline characteristics of patients stratified by levels of proteinuria.

	Proteinuria (dipstick)				
	negative/trace	1+	2+	3+	
Variable	n = 48	n = 137	n = 430	n = 765	P for trend
Age (years)	75 ± 11	71 ± 12	68 ± 13	66 ± 13	0.12
Male (%)	31 (65%)	83 (61%)	282 (66%)	537 (70%)	0.64
BMI (kg/m^2^)	21.5 ± 4.0	22.4 ± 3.7	22.9 ± 4.1	24.2 ± 4.4	0.04
Comorbidities					
Diabetes mellitus (%)	22 (46%)	45 (33%)	166 (39%)	473 (62%)	0.95
History of coronary artery disease (%)	14 (29%)	28 (20%)	42 (10%)	77 (10%)	<0.001
Heart failure (%)	20 (42%)	33 (24%)	79 (18%)	143 (19%)	0.04
Stroke (%)	9 (19%)	31 (23%)	63 (15%)	113 (15%)	0.04
Barthel index	71 ± 30	84 ± 27	87 ± 25	90 ± 21	<0.001
Primary Disease					
Diabetic nephropathy (%)	8 (17%)	33 (24%)	130 (30%)	429 (56%)	0.01
Nephrosclerosis (%)	28 (58%)	52 (38%)	128 (30%)	140 (18%)	<0.001
Chronic glomerulonephritis (%)	2 (4%)	13 (9%)	84 (20%)	105 (14%)	<0.001
Polycystic kidney disease (%)	2 (4%)	16 (12%)	18 (4%)	8 (1%)	<0.001
Other or unknown (%)	8 (17%)	23 (17%)	70 (16%)	83 (11%)	<0.001
Oral medication					
Use of ACEI/ARB (%)	29 (60%)	80 (58%)	251 (58%)	473 (62%)	0.98
Use of diuretics (%)	36 (75%)	98 (72%)	285 (66%)	546 (71%)	0.71
SBP (mm Hg)	129 ± 31	141 ± 26	146 ± 24	158 ± 24	0.09
DBP (mm Hg)	66 ± 18	72 ± 14	76 ± 14	80 ± 15	0.21
eGFR (mL/min/1.73 m^2^)	8.2 ± 3.9	5.7 ± 2.4	5.4 ± 1.8	5.2 ± 1.9	<0.001
Serum albumin (g/dL)	3.3 ± 0.6	3.4 ± 0.6	3.3 ± 0.6	3.1 ± 0.6	0.42
Total cholesterol (mg/dL)	142 ± 42	150 ± 40	156 ± 43	168 ± 45	0.36
Hemoglobin (g/dL)	9.5 ± 1.7	9.4 ± 1.5	9.3 ± 1.6	9.4 ± 1.5	0.17
WBC (1,000/mm^3^)	6.1 ± 2.4	6.2 ± 2.3	6.4 ± 3.7	6.9 ± 3.0	<0.001
Ejection Fraction (%)	54.8 ± 17.4	59.9 ± 14.3	60.7 ± 12.5	61.5 ± 10.8	<0.001

Values are mean ± SD, % of the total. BMI body mass index, ACEI Angiotensin-converting-enzyme inhibitor, ARB Angiotensin Receptor Blockers, SBP systolic blood pressure, DBP diastolic blood pressure, eGFR estimated glomerular filtration rate, WBC white blood cell

### Associations of proteinuria with mortality risk

**Fig 1:**
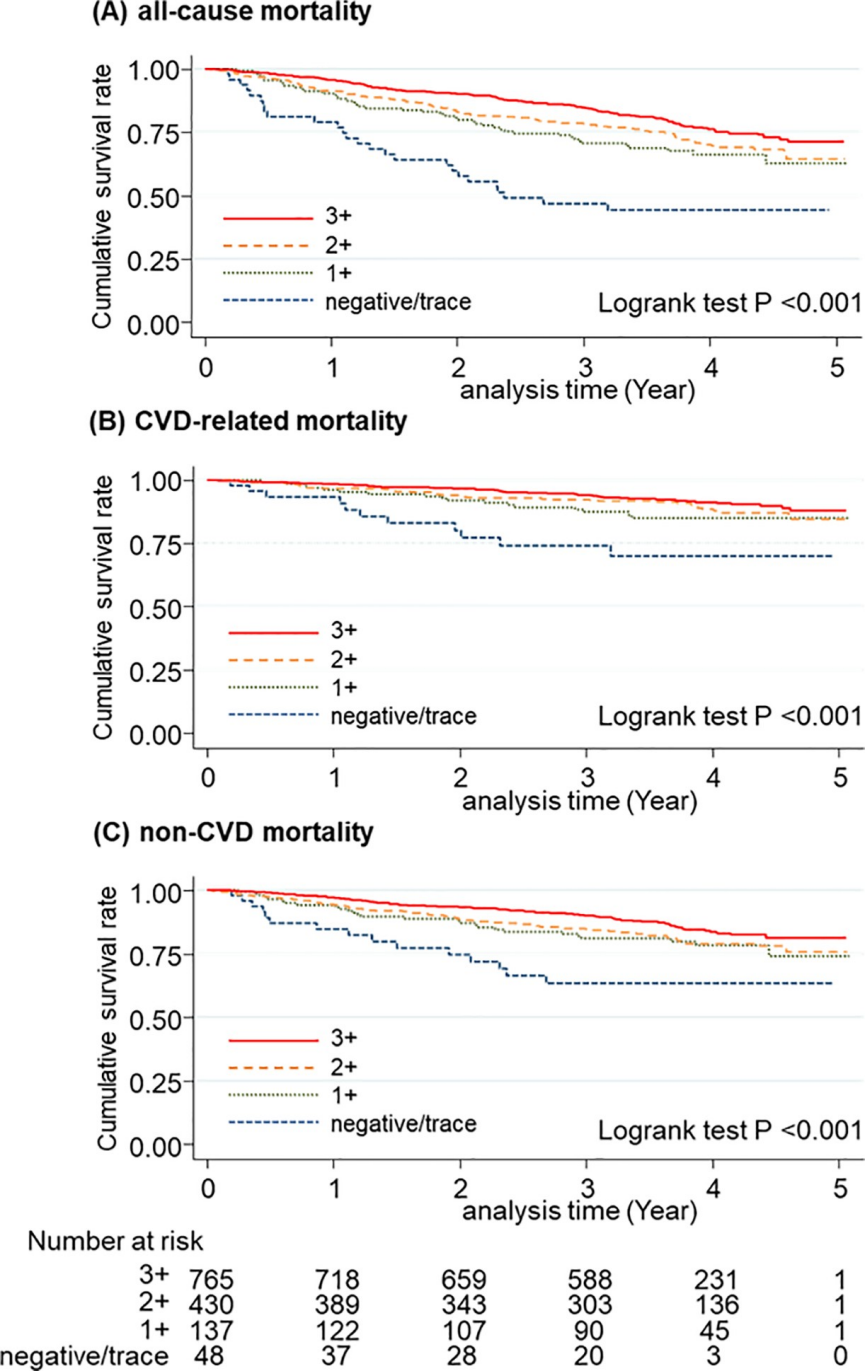
Survival estimates for all-cause mortality (A), CVD mortality (B), and non-CVD mortality (C) according to dipstick proteinuria categories (negative/trace, 1+, 2+, and ≥3+).

During a median follow-up of 3.5 (IQI 2.8–4.2) years, 352 patients died (129 deaths due to CVD and 223 deaths due to non-CVD causes). Fig 1 shows survival estimates across the four categories of dipstick proteinuria. As hypothesized, the dipstick proteinuria negative/trace had the worst prognosis. Approximately half of the patients in this category died in the first two years (Fig 1A). The dipstick proteinuria category 1+ also demonstrated worse prognosis compared to the categories 2+ and ≥3+. Similar results were found for both CVD mortality and non-CVD mortality (Fig 1B and 1C).

Higher mortality risk in negative/trace dipstick proteinuria, compared to dipstick proteinuria ≥3+, remained significant even after accounting for demographic (Model 2 in Table 2) and clinical factors (Models 3 and 4). Specifically, negative/trace dipstick proteinuria conferred ~3-fold higher risk of mortality in these three multivariable models (e.g., adjusted hazard ratio 2.60 [95% CI, 1.62–4.17] in Model 4). Both dipstick proteinuria 1+ and 2+ were significantly associated with a higher risk of all-cause mortality in Model 1 (Table 2) as well. Although these associations were attenuated in Models 2–4, the association for dipstick proteinuria 1+ was borderline significant (e.g., p = 0.1 in Model 4). Nonetheless, p for trend was consistently <0.001 in all Models 1–4. Generally, similar results were observed for CVD mortality and non-CVD mortality (Table 2) although hazard ratios appeared slightly greater for CVD mortality than non-CVD mortality in Models 1–3 (but not necessarily in Model 4).

**Table 2:** HRs (95% CI) of mortality outcomes according to dipstick proteinuria categories.

	Proteinuria (dipstick)				
	negative/trace	1+	2+	≥3+	P for trend
	(n = 48)	(n = 137)	(n = 430)	(n = 765)	(n = 1380)
**All-cause mortality (352 deaths)**	26 deaths	44 deaths	119 deaths	163 deaths	
Model 1	3.86 (2.55–5.85) **	1.62 (1.16–2.26) **	1.35 (1.07–1.71) *	ref	<0.001
Model 2	3.07 (2.02–4.65) **	1.37 (0.98–1.92)	1.25 (0.98–1.58)	ref	<0.001
Model 3	2.84 (1.86–4.33) **	1.32 (0.93–1.86)	1.27 (1.00–1.61)	ref	<0.001
Model 4	2.60 (1.62–4.17) **	1.35 (0.95–1.93)	1.25 (0.97–1.62)	ref	<0.001
**CVD mortality (129 deaths)**	11 deaths	17 deaths	41 deaths	60 deaths	
Model 1	4.47 (2.35–8.52) **	1.71 (0.99–2.92)	1.27 (0.85–1.89)	ref	<0.001
Model 2	3.76 (1.97–7.19) **	1.52 (0.89–2.62)	1.20 (0.81–1.79)	ref	0.001
Model 3	3.23 (1.67–6.25) **	1.56 (0.89–2.73)	1.30 (0.86–1.94)	ref	0.001
Model 4	2.44 (1.13–5.26) *	1.42 (0.80–2.54)	1.17 (0.76–1.80)	ref	0.03
**non-CVD mortality (223 deaths)**	15 deaths	27 deaths	78 deaths	103 deaths	
Model 1	3.51 (2.04–6.04) **	1.57 (1.02–2.39) *	1.40 (1.04–1.88) *	ref	<0.001
Model 2	2.70 (1.57–4.66) **	1.29 (0.84–1.97)	1.27 (0.95–1.71)	ref	0.002
Model 3	2.63 (1.51–4.56) **	1.22 (0.79–1.89)	1.25 (0.93–1.69)	ref	0.005
Model 4	2.67 (1.45–4.92) **	1.36 (0.86–2.13)	1.31 (0.95–1.81)	ref	0.004

Model1; unadjusted, Model2; adjusted for age, gender, Model3; Model2+ diabetes mellitus, coronary artery disease, heart failure, stroke, Model4; Model3+ systolic blood pressure, total cholesterol, eGFR, serum albumin, hemoglobin, white blood cell, diuretics, angiotensin-converting-enzyme inhibitor/angiotensin receptor blockers

* P < 0.05

** P < 0.01

### Sensitivity analysis

The associations were largely consistent when we restricted our analysis to patients aged 70 years or older (Table 3). The hazard ratio for negative/trace dipstick proteinuria (vs. ≥3+) ranged from 2.8 to 3.7 across Models 1–4. Dipstick proteinuria 1+ remained significant up to Model 3 in this sensitivity analysis (adjusted hazard ratio 1.45 [95%CI 1.00–2.10] in Model 3). All four models demonstrated a dose-response relationship between lower proteinuria with higher all-cause mortality risk, with all p for trend ≤0.001. The patterns were similar for CVD mortality and non-CVD mortality, but again, the associations appeared stronger for CVD mortality than non-CVD mortality. Similarly, results were consistent when we investigated patients with nephrosclerosis (S1 Table). Of note, in this analysis, even dipstick proteinuria 1+ remained significant for all-cause mortality up to Model 4 (2.36 [95% CI 1.32–4.22]).

**Table 3:** HRs (95% CI) of mortality outcomes according to dipstick proteinuria categories in patients aged 70 years or older.

	Proteinuria (dipstick)				
	negative/trace	1+	2+	≥3+	P for trend
	(n = 36)	(n = 88)	(n = 233)	(n = 339)	(n = 696)
**All-cause mortality (262 deaths)**	24 deaths	40 deaths	89 deaths	109 deaths	
Model 1	3.68 (2.36–5.73) **	1.55 (1.08–2.23) *	1.26 (0.95–1.66)	ref	<0.001
Model 2	3.54 (2.27–5.52) **	1.54 (1.07–2.22) *	1.27 (0.96–1.68)	ref	<0.001
Model 3	3.37 (2.15–5.30) **	1.45 (1.00–2.10) *	1.26 (0.95–1.68)	ref	<0.001
Model 4	2.84 (1.70–4.76) **	1.39 (0.95–2.05)	1.14 (0.84–1.55)	ref	0.001
**CVD mortality (86 deaths)**	10 deaths	15 deaths	31 deaths	30 deaths	
Model 1	5.66 (2.76–11.61) **	2.14 (1.15–3.98) *	1.61 (0.97–2.65)	ref	<0.001
Model 2	5.56 (2.70–11.43) **	2.15 (1.15–3.99) *	1.62 (0.98–2.67)	ref	<0.001
Model 3	4.92 (2.36–10.28) **	2.03 (1.07–3.86) *	1.64 (0.99–2.72)	ref	<0.001
Model 4	2.97 (1.28–6.87) *	1.65 (0.84–3.21)	1.27 (0.74–2.19)	ref	0.01
**non-CVD mortality (176 deaths)**	14 deaths	25 deaths	58 deaths	79 deaths	
Model 1	2.94 (1.66–5.20) **	1.33 (0.85–2.09)	1.13 (0.80–1.58)	ref	0.003
Model 2	2.81 (1.58–4.97) **	1.31 (0.84–2.06)	1.14 (0.81–1.60)	ref	0.004
Model 3	2.76 (1.55–4.94) **	1.24 (0.78–1.97)	1.12 (0.80–1.58)	ref	0.009
Model 4	2.83 (1.46–5.47) **	1.30 (0.80–2.10)	1.11 (0.77–1.60)	ref	0.01

Model1; unadjusted, Model2; adjusted for age, gender, Model3; Model2+ diabetes mellitus, coronary artery disease, heart failure, stroke, Model4; Model3+ systolic blood pressure, total cholesterol, eGFR, serum albumin, hemoglobin, white blood cell, diuretics, angiotensin-converting-enzyme inhibitor/angiotensin receptor blockers

* P < 0.05

** P < 0.01

When we additionally adjusted for ejection fraction, the associations were slightly attenuated but largely remained consistent (Table 4). The patterns were similar when we restricted this model with ejection fraction to patients aged 70 years or older.

**Table 4:** HRs (95% CI) of mortality outcomes according to dipstick proteinuria categories (additionally adjusted for ejection fraction) in overall and 70 years or older.

	Proteinuria (dipstick)				
	negative/trace	1+	2+	≥3+	P for trend
**(A) overall**	**(n = 45)**	**(n = 118)**	**(n = 354)**	**(n = 625)**	**(n = 1142)**
All-cause mortality (286 deaths)	24 deaths	40 deaths	89 deaths	133 deaths	
	2.55 (1.55–4.18) **	1.35 (0.92–1.99)	1.09 (0.82–1.46)	ref	0.002
CVD mortality (108 deaths)	10 deaths	16 deaths	32 deaths	50 deaths	
	2.30 (1.02–5.20) *	1.47 (0.79–2.74)	1.01 (0.62–1.63)	ref	0.06
non-CVD mortality (178 deaths)	14 deaths	24 deaths	57 deaths	83 deaths	
	2.64 (1.40–4.99) **	1.34 (0.82–2.21)	1.15 (0.80–1.66)	ref	0.01
**(B) 70 years or older**	(n = 35)	(n = 79)	(n = 193)	(n = 286)	(n = 593)
All-cause mortality (214 deaths)	23 deaths	36 deaths	65 deaths	90 deaths	
	2.70 (1.58–4.63) **	1.34 (0.88–2.04)	0.98 (0.69–1.38)	ref	0.003
CVD mortality (74 deaths)	10 deaths	14 deaths	24 deaths	26 deaths	
	3.17 (1.33–7.58) *	1.70 (0.83–3.49)	1.12 (0.62–2.05)	ref	0.01
non-CVD mortality (140 deaths)	13 deaths	22 deaths	41 deaths	64 deaths	
	2.45 (1.22–4.92) *	1.20 (0.71–2.03)	0.91 (0.60–1.41)	ref	0.06

Model; Model4+ ejection fraction

* P < 0.05

** P < 0.01

**Fig 2:**
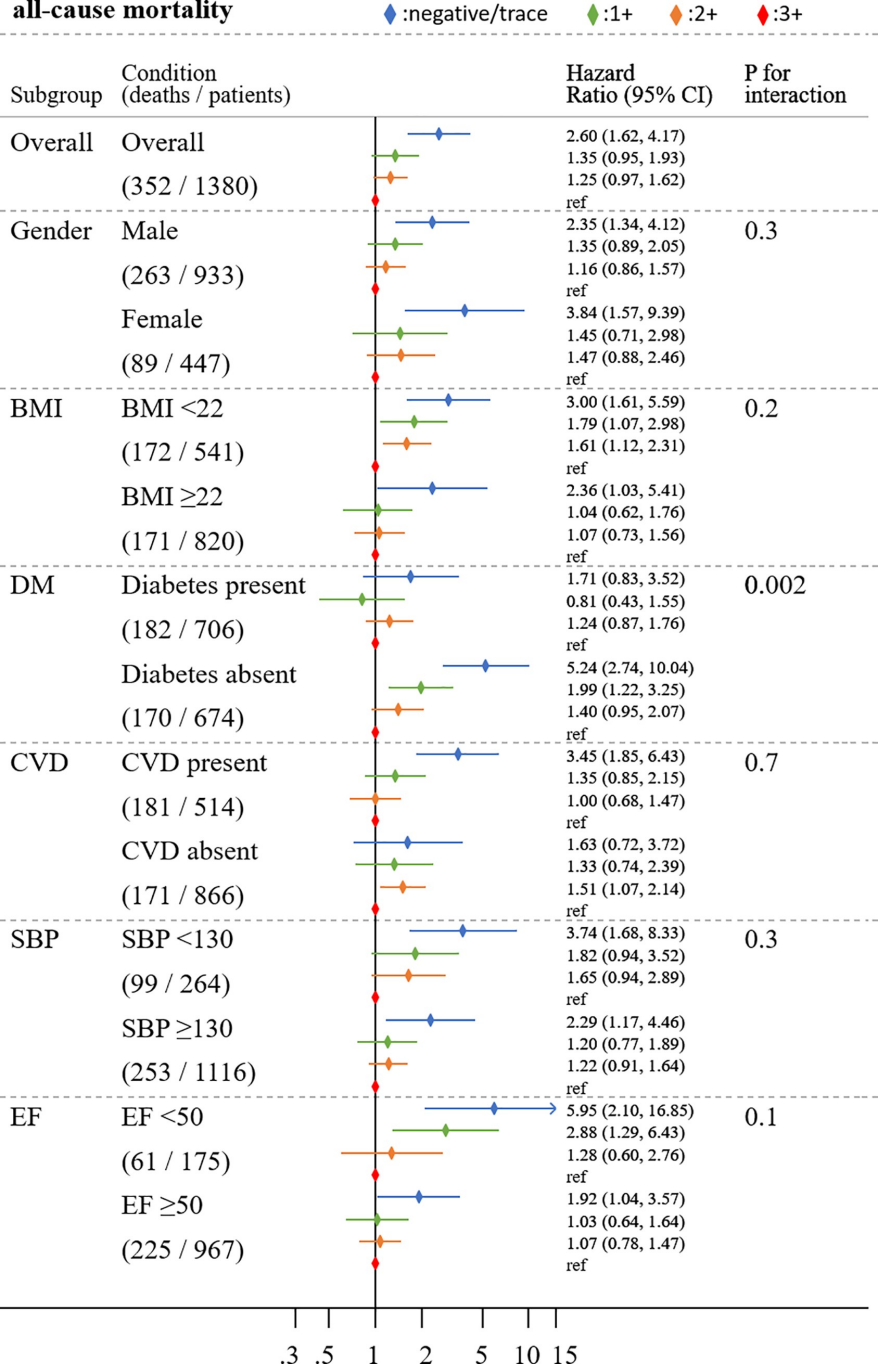
Adjusted HRs (95% CI) of all-cause mortality according to four dipstick proteinuria categories across major subgroups.

The association of proteinuria with all-cause mortality was largely similar across demographics and clinical subgroups tested (Fig 2). Although we observed a few significant interactions for specific mortality outcomes (gender for CVD mortality and BMI for non-CVD mortality [S2 and S3 Figs]), diabetic status was the only factor with a significant interaction in all-cause mortality (p for interaction 0.002) as well as non-CVD mortality (p for interaction 0.001), with a stronger association in non-diabetes than in diabetes. For ejection fraction, the results were more evident among patients with lower ejection fraction than those with preserved ejection fraction, but its interaction with proteinuria did not reach statistical significance.

Adjusted for age, gender, diabetes mellitus, coronary artery disease, heart failure, stroke, systolic blood pressure (SBP) total cholesterol, diuretics, angiotensin-converting-enzyme inhibitor/angiotensin receptor blockers, estimated glomerular filtration rate (eGFR), serum albumin, hemoglobin, white blood cell (WBC). The P values represent significance levels for interaction terms. BMI = body mass index; CVD = cardiovascular disease; EF = ejection fraction.

## Discussion

Current cohort, AICOPP is unique in terms of connecting the clinical condition at the time of dialysis initiation with the prognosis after the initiation. From AICOPP, several articles have been published. The higher prevalence of heart failure related to symptoms at the time of dialysis initiation and the poorer survival prognosis observed in those with rapid eGFR decline in the three months prior to dialysis initiation [14], and elevated mortality risk associated with the presence of atrial fibrillation at the time of dialysis initiation [15] are findings from this cohort.

Among incident dialysis patients in AICOPP, as hypothesized, we observed an increased risk of mortality along with lower levels of proteinuria. The hazard ratio of mortality outcomes for negative/trace dipstick proteinuria (vs. ≥3+) was ~2.5 to ~3 after accounting for a number of potential confounders. Although the prevalence of negative/trace dipstick proteinuria was low in this clinical population, we generally observed a dose-response relationship across four categories of dipstick proteinuria. Importantly, this inverse association of proteinuria with mortality risk was robust even after accounting for key factors related to negative/trace dipstick in this clinical population (i.e., older age and nephrosclerosis as primary kidney disease). Also, the results were largely consistent across demographic and clinical subgroups (although a weaker association in diabetes vs. non-diabetes) and between CVD mortality and non-CVD mortality.

Although counterintuitive, our finding of elevated risk of mortality according to lower levels of proteinuria is consistent with reports of a J-shaped association between proteinuria and mortality from the CKD Prognosis Consortium as well as US veterans in patients with eGFR <30 ml/min/1.73m^2^ [6, 9]. Our results were even more extreme than those two previous reports since we observed an inverse, instead of a J-shaped, association between proteinuria and mortality. Potential reasons for the difference in findings between our study and the previous reports may reflect different study populations (ours were Japanese and all required dialysis) and/or design (our study used semi-quantitative measure of proteinuria using dipstick strips). Nonetheless, to the best of our knowledge, our study is the first to describe in detail the association of low proteinuria with increased risk of clinical outcomes specifically in patients with incident dialysis.

Potential reasons for our findings warrant discussion. First, there is the potential for confounding, with differences in patient characteristics by level of proteinuria. Specifically, patients with lower levels of proteinuria were older and tended to have a higher prevalence of CVD, compared to those with higher proteinuria. Also, nephrosclerosis was a major kidney disease in patients with low levers of proteinuria. However, the results were robust even after accounting for these factors. Second, low proteinuria may be an indicator of low organ perfusion in this specific population. Indeed, patients with lower proteinuria had lower blood pressure in our analysis, and lower blood pressure is associated with poor prognosis in patients on dialysis [16, 17]. Nonetheless, lower proteinuria was related to poor prognosis in patients with higher blood pressure in our study as well. Also, the association of lower proteinuria with higher mortality was independent of cardiac ejection fraction. A third possibility is that low proteinuria may reflect low urine concentration by impaired kidney function or use of diuretics. Although we accounted for diuretic use in our analysis, our study did not have data on urine volume and urine creatinine concentration to explore this possibility.

The association between lower levels of proteinuria and increased mortality risk was less evident in diabetes than in non-diabetes, particularly for non-CVD mortality. Diabetes is a leading cause of proteinuria, and a number of previous studies demonstrated that elevated proteinuria is particularly prognostic in patients with diabetes [7, 18]. Thus, it is possible that excess risk related to low proteinuria in patients with incident dialysis might not be evident in the presence of diabetes since those with diabetes and high proteinuria also have high mortality risk. The particular interaction for non-CVD mortality may be related to the observation that proteinuria is associated with the risk of infection in patients with diabetes mellitus [19, 20] and infection is the most frequent cause of non-CVD mortality in Japan [21]. Nonetheless, we should interpret this interaction due to diabetic status as hypothesis-generating since we tested multiple subgroups without a priori hypothesis.

Our study has clinical and research implications. Regardless of the reason for our findings, healthcare providers, particularly nephrologists, should be aware that low proteinuria is associated with poor prognosis in patients with severely reduced GFR. This is crucial since according to the KDIGO CKD clinical practice guideline, information on proteinuria is routinely collected in patients with kidney disease. Additionally, future investigations need to be done to characterize trajectories of proteinuria before and after the development of severely reduced GFR (for example, those with low proteinuria might have high proteinuria before they reached the stage of severely reduced eGFR).

In addition to a lack of data on urine volume, our study has limitations. First, although proteinuria is known to have a relatively large short-term variation, we have only a single assessment of proteinuria [1]. Nonetheless, misclassification as a result of imprecision usually leads to null associations. Second, as discussed above, proteinuria was based on semi-quantitative assessment with dipstick strips, which is not able to account for urine concentration. Thus, future studies in dialysis patients are needed to explore this study question with urine albumin- or protein-to-creatinine ratio. Still, our study is quite relevant because dipstick assessment of proteinuria is simple, inexpensive, and thus, routinely used and interpreted in clinical practice [5, 22]. Finally, our study only included Japanese patients. Thus, confirmatory studies are important in incident dialysis patients from other regions/countries and other racial/ethnic groups.

In conclusion, lower levels of pre-dialysis proteinuria were significantly associated with a higher risk of mortality in incident dialysis patients. This association was robust even when adjusted for several potential confounders and consistent across subgroups. Future studies are needed to examine potential reasons for our observations. Nonetheless, our findings highlight the need for healthcare providers, particularly nephrologists, to carefully interpret proteinuria data among incident dialysis patients.

## Supporting information

S1 Fig Flow chart for patient selection. (TIF)

S2 Fig Adjusted HRs (95% CI) of CVD mortality outcomes according to four dipstick proteinuria categories across major subgroups. (TIF)

S3 Fig Adjusted HRs (95% CI) of non-CVD mortality outcomes according to four dipstick proteinuria categories across major subgroups. (TIF)

S1 Table HRs (95% CI) of mortality outcomes according to dipstick proteinuria categories in nephrosclerosis patients. Model1; unadjusted,
Model2; adjusted for age, gender,
Model3; Model2+ diabetes mellitus, coronary artery disease, heart failure, stroke,
Model4; Model3+ systolic blood pressure, total cholesterol, eGFR, serum albumin, hemoglobin, white blood cell, diuretics, angiotensin-converting-enzyme inhibitor/angiotensin receptor blockers
* P < 0.05, ** P < 0.01.
(DOCX)
