# Supplementary figures and images for: Lower levels of proteinuria are associated with elevated mortality in incident dialysis patients
Source: PLoS One. 2019 Dec 23;14(12):e0226866. doi: 10.1371/journal.pone.0226866 (PMC6927646; doi:10.1371/journal.pone.0226866)

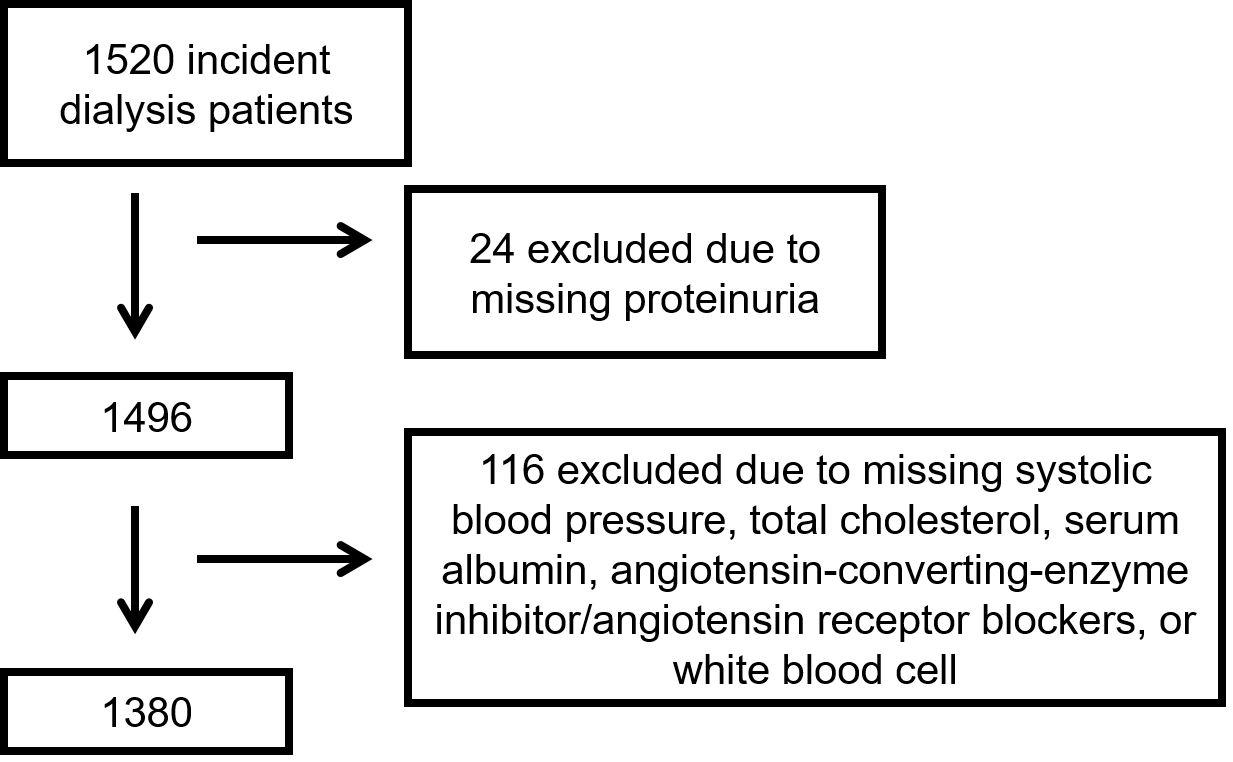

Supplement: S1 Fig — (TIF) [file pone.0226866.s001.tif]

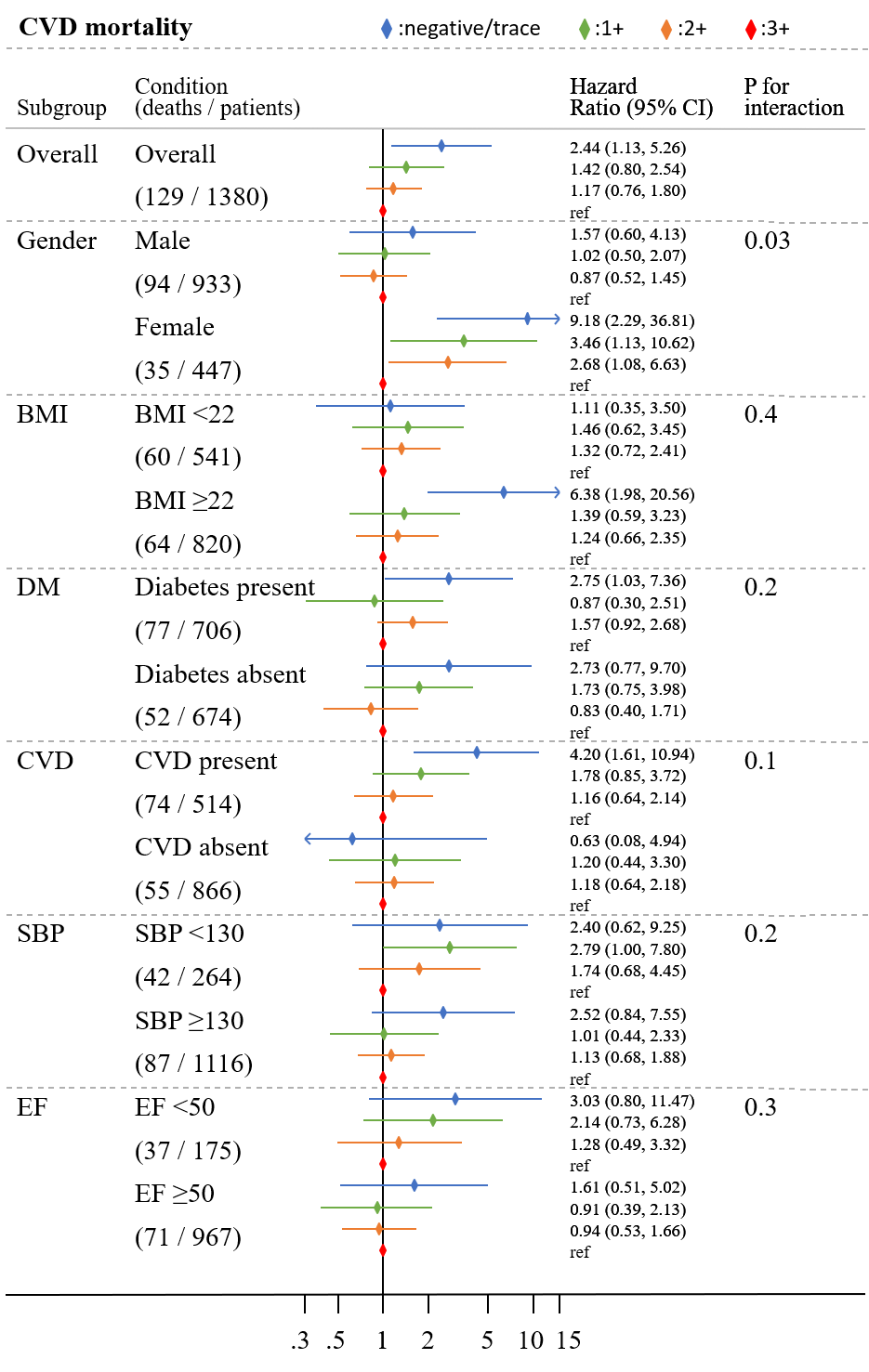

Supplement: S2 Fig — (TIF) [file pone.0226866.s002.tif]

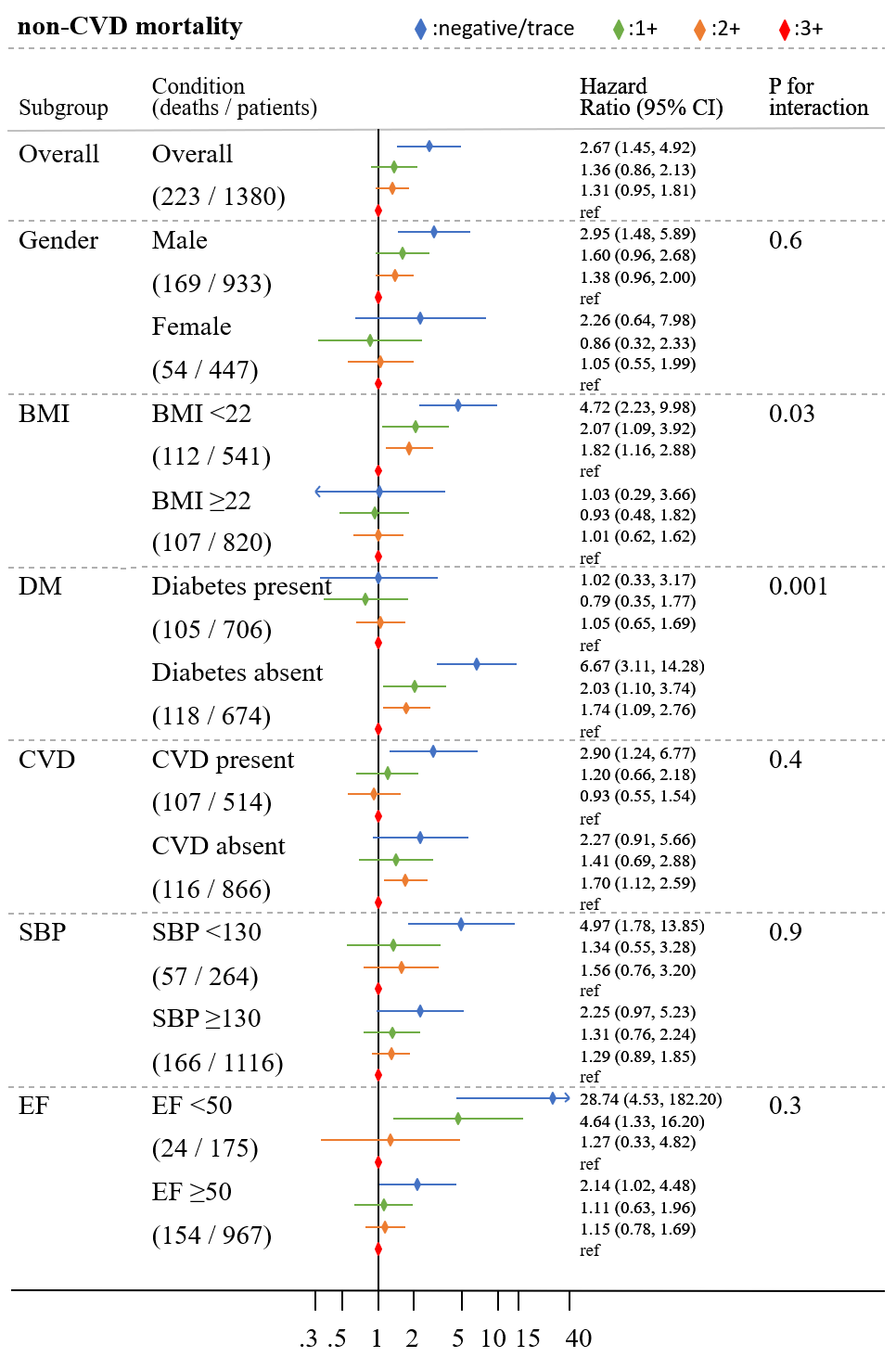

Supplement: S3 Fig — (TIF) [file pone.0226866.s003.tif]
