# Supplementary material for: Lower levels of proteinuria are associated with elevated mortality in incident dialysis patients
Source: PLoS One. 2019 Dec 23;14(12):e0226866. doi: 10.1371/journal.pone.0226866 (PMC6927646; doi:10.1371/journal.pone.0226866)
Supplement: S1 Table — Model1; unadjusted, Model2; adjusted for age, gender, Model3; Model2+ diabetes mellitus, coronary artery disease, heart failure, stroke, Model4; Model3+ systolic blood pressure, total cholesterol, eGFR, serum albumin, hemoglobin, white blood cell, diuretics, angiotensin-converting-enzyme inhibitor/angiotensin receptor blockers * P < 0.05, ** P < 0.01. (DOCX) [file pone.0226866.s004.docx]

|  | **Proteinuria (dipstick)** | | | |  |
| --- | --- | --- | --- | --- | --- |
|  | **negative/trace** | **1+** | **2+** | **3+** | **P for trend** |
|  | **(n=28)** | **(n=52)** | **(n=128)** | **(n=140)** | **(n=348)** |
| **All-cause mortality (112 deaths)** | 17 deaths | 27 deaths | 41 deaths | 37 deaths |  |
| Model 1 | 4.50 (2.52-8.03) ** | 2.54 (1.54-4.17) ** | 1.21 (0.78-1.89) | ref | <0.001 |
| Model 2 | 5.22 (2.90-9.40) ** | 2.63 (1.59-4.33) ** | 1.32 (0.84-2.07) | ref | <0.001 |
| Model 3 | 5.01 (2.69-9.36) ** | 2.38 (1.40-4.07) ** | 1.25 (0.80-1.98) | ref | <0.001 |
| Model 4 | 5.58 (2.54-12.28) ** | 2.36 (1.32-4.22) * | 1.19 (0.73-1.95) | ref | <0.001 |
| **CVD mortality (41 deaths)** | 6 deaths | 12 deaths | 12 deaths | 11 deaths |  |
| Model 1 | 5.28 (1.94-14.37) ** | 3.83 (1.69-8.69) ** | 1.19 (0.53-2.71) | ref | <0.001 |
| Model 2 | 5.62 (2.04-15.49) ** | 3.92 (1.72-8.93) ** | 1.25 (0.55-2.85) | ref | <0.001 |
| Model 3 | 5.71 (1.95-16.73) ** | 3.66 (1.53-8.75) ** | 1.14 (0.50-2.64) | ref | <0.001 |
| Model 4 | 3.73 (0.98-14.27) | 2.59 (0.99-6.78) | 0.88 (0.36-2.16) | ref | 0.02 |
| **non-CVD mortality (81 deaths)** | 11 deaths | 15 deaths | 29 deaths | 26 deaths |  |
| Model 1 | 4.17 (2.05-8.48) ** | 2.00 (1.06-3.77) * | 1.22 (0.72-2.07) | ref | <0.001 |
| Model 2 | 5.14 (2.49-10.61) ** | 2.07 (1.09-3.93) * | 1.37 (0.80-2.33) | ref | <0.001 |
| Model 3 | 4.66 (2.16-10.08) ** | 1.85 (0.93-3.67) | 1.31 (0.76-2.25) | ref | <0.001 |
| Model 4 | 7.35 (2.74-19.70) ** | 2.21 (1.05-4.67) * | 1.40 (0.78-2.52) | ref | 0.001 |
